# Structural modeling of GSK3β implicates the inactive (DFG-*out*) conformation as the target bound by TDZD analogs

**DOI:** 10.1038/s41598-020-75020-w

**Published:** 2020-10-27

**Authors:** Meenakshisundaram Balasubramaniam, Nirjal Mainali, Suresh Kuarm Bowroju, Paavan Atluri, Narsimha Reddy Penthala, Srinivas Ayyadevera, Peter A. Crooks, Robert J. Shmookler Reis

**Affiliations:** 1 McClellan Veterans Medical Ctr., Central Arkansas Veterans Healthcare Service, Little Rock, AR 72205 USA; 2 Department of Geriatrics, Reynolds Institute on Aging, University of Arkansas for Medical Sciences, Little Rock, AR 72205 USA; 3 Bioinformatics Program, University of Arkansas for Medical Sciences, and University of Arkansas At Little Rock, Little Rock, AR 72205 USA; 4 Summer Research Internship Program, University of Arkansas for Medical Sciences, Little Rock, AR 72205 USA; 5 Department of Pharmaceutical Sciences, College of Pharmacy, University of Arkansas for Medical Sciences, Little Rock, AR 72205 USA

**Keywords:** Biochemistry, Computational biology and bioinformatics, Drug discovery, Molecular biology, Molecular medicine

## Abstract

Glycogen synthase kinase-3β (GSK3β) controls many physiological pathways, and is implicated in many diseases including Alzheimer’s and several cancers. GSK3β-mediated phosphorylation of target residues in microtubule-associated protein tau (MAPTAU) contributes to MAPTAU hyperphosphorylation and subsequent formation of neurofibrillary tangles. Inhibitors of GSK3β protect against Alzheimer’s disease and are therapeutic for several cancers. A thiadiazolidinone drug, TDZD-8, is a non-ATP-competitive inhibitor targeting GSK3β with demonstrated efficacy against multiple diseases. However, no experimental data or models define the binding mode of TDZD-8 with GSK3β, which chiefly reflects our lack of an established inactive conformation for this protein. Here, we used metadynamic simulation to predict the three-dimensional structure of the inactive conformation of GSK3β. Our model predicts that phosphorylation of GSK3β Serine9 would hasten the DFG-flip to an inactive state. Molecular docking and simulation predict the TDZD-8 binding conformation of GSK3β to be inactive, and are consistent with biochemical evidence for the TDZD-8–interacting residues of GSK3β. We also identified the pharmacophore and assessed binding efficacy of second-generation TDZD analogs (TDZD-10 and Tideglusib) that bind GSK3β as non-ATP-competitive inhibitors. Based on these results, the predicted inactive conformation of GSK3β can facilitate the identification of novel GSK3β inhibitors of high potency and specificity.

## Introduction

Glycogen synthase kinase-3 beta (GSK3β) is a serine/threonine protein kinase expressed ubiquitously in mammalian cells. GSK3β regulates many physiological pathways by phosphorylating key downstream targets. The kinase activity of GSK3β is implicated in many cancers and in neurodegenerative diseases, including Alzheimer’s and Parkinson’s diseases^1–3^. For instance, GSK3β-mediated hyperphosphorylation of two key residues, Ser202 and Thr205, of the microtubule-associated protein tau (MAPT or TAU)^4^ alters microtubule structure and function. This contributes critically to the formation of hyperphosphorylated tau (hP-tau) and initiates the accrual of paired helical filaments that comprise the neurofibrillary tangles (NFTs), which are specifically recognized by antibody AT8^5^. The presence and high abundance of such tangle-bearing neurons is a neuropathological hallmark of Alzheimer’s disease^6^. Hyperphosphorylation of tau, triggered by either MAPK-p38 as previously reported^7^ or by GSK3β as shown here, is associated with decreased interactions of tau with microtubules and results in destabilization of microtubules and subsequent decrease in the efficient transport of organelles, proteins, etc., within the neuron^8^.

GSK3β resembles other serine/threonine protein kinases in structure, showing a conserved catalytic domain comprising an *N*-terminal glycine-rich loop region that is connected by a “hinge” to a C-terminal lobe^9,10^. A highly conserved loop within the C-terminal domain, termed the “activation loop,” contains an invariant “DFG” motif (Asp-Phe-Gly), which plays a key role in determining kinase activity. Based on the structural orientation of this conserved Phe and Asp, protein kinases can be distinguished in two conformations, i.e., “active” (“DFG-*in*”) or “inactive” (“DFG-*out*”)^9,11^. When the kinase is in its active conformation, mostly determined by phosphorylation of a tyrosine residue in the activation loop, ATP (adenosine triphosphate) binds to the conserved active-site pocket between the *N*-terminal glycine-rich loop and the C-terminal lobe^12^. The first crystal structure of an inactive kinase conformation was determined for tyrosine-protein kinase Src (c-Src), a proto-oncogene. This structure showed Asp and Phe residues rotated ~ 180° relative to their orientations in the active conformation, a flip shared by several other kinases in their crystallography-based inactive conformations^9^. Active and inactive conformations of all kinases are determined primarily by the orientations of Asp (D) and Phe (F) sidechains in the DFG motif^12^. In the active state, Phe in the activation-loop DFG extends its sidechain inward (toward the αC-helix at the *N*-terminus), while the Asp sidechain faces outward (away from the αC-helix); this motif orientation is denoted as “DFG-*in*”^13^. In the inactive conformation, Phe flips to face outward while Asp rotates to face inward, denoted as “DFG-*out*”^11,13^. This DFG flip creates an allosteric hydrophobic pocket in the inactive state, adjacent to the ATP-binding region^9,11^.

Several potent kinase inhibitors, including imatinib, have been reported to bind to kinases that are in their inactive conformation. Energetically, kinase inhibition can be most efficiently achieved by stabilizing the allosteric pocket of the inactive conformation (ATP-noncompetitive inhibitors), whereas inhibitors that block the ATP-binding pocket (ATP-competitive inhibitors) must compete with an abundant supply of endogenous ATP^9,11,14^. Therefore, accurate modeling of a target kinase’s inactive conformation is crucial for the design of target-specific inhibitors. Since kinases oscillate between active and inactive conformations, elucidating experimental models is challenging.

GSK3β is thought to be constitutively active in its native form, and is inactivated upon cell stimulation by insulin^15^. Scarcity of the inactive form, and the inherent structural instability of its activation loop, pose major obstacles to molecular modeling based on X-ray crystallography. Notwithstanding its allegedly constitutive activity, the presence of DFG residues in the GSK3β activation loop suggests a potential for inactivation via a DFG-flip. Phosphorylation of Ser9 was shown to inactivate GSK3β^16^, and to inhibit substrate binding^17,18^, leading us to speculate that this phosphorylation may induce a DFG flip.

Selective ATP-noncompetitive inhibitors, including TDZD-8, were proposed to act at the allosteric hydrophobic pocket of GSK3β^19,20^, which is only present in the inactive state. However, the mechanism by which known GSK3β ATP-noncompetitive inhibitors bind to the allosteric pocket of GSK3β remains to be elucidated. Mechanistic studies have been hindered by the lack of a structural model for GSK3β in its inactive conformation, impeding drug development by the usual methods, i.e., lead optimization based on the binding mode of a parent compound such as TDZD-8. For example, the TDZD analog Tideglusib is a potent ATP-noncompetitive GSK3β inhibitor. Tideglusib is known to inhibit GSK3β irreversibly, suggesting the possibility of covalent interaction between the drug (Tideglusib) and the protein (GSK3β)^20^. However, in the absence of an experimental structure for GSK3β binding to Tideglusib, it has not been possible to visualize or identify the site of covalent bond formation. Since Tidglusib is a TDZD analog and is proposed to act as ATP-noncompetitive inhibitor^20^, it is expected to bind to the allosteric pocket of GSK3β. Without structural details, exemplifying binding modes of potent compounds is tedious. Defining the binding mode of inhibitors such as Tideglusib is crucial to understanding its mechanism of action, its pharmacophore, and the key residues of GSK3β involved. This pivotal information is required to develop potent second-generation drugs, capable of inhibiting GSK3β specifically with minimal off-target effects. We now propose a structure for GSK3β in the inactive DFG-*out* conformation, which has been derived from metadynamic-simulation modeling. Several validation procedures support this predicted inactive conformation of GSK3β, which can be retrieved from this site: https://data.mendeley.com/datasets/d69pzg3syh/1.

## Results

### Metadynamic-simulation modeling predicts the inactive conformation of GSK3β

**Figure 1:**
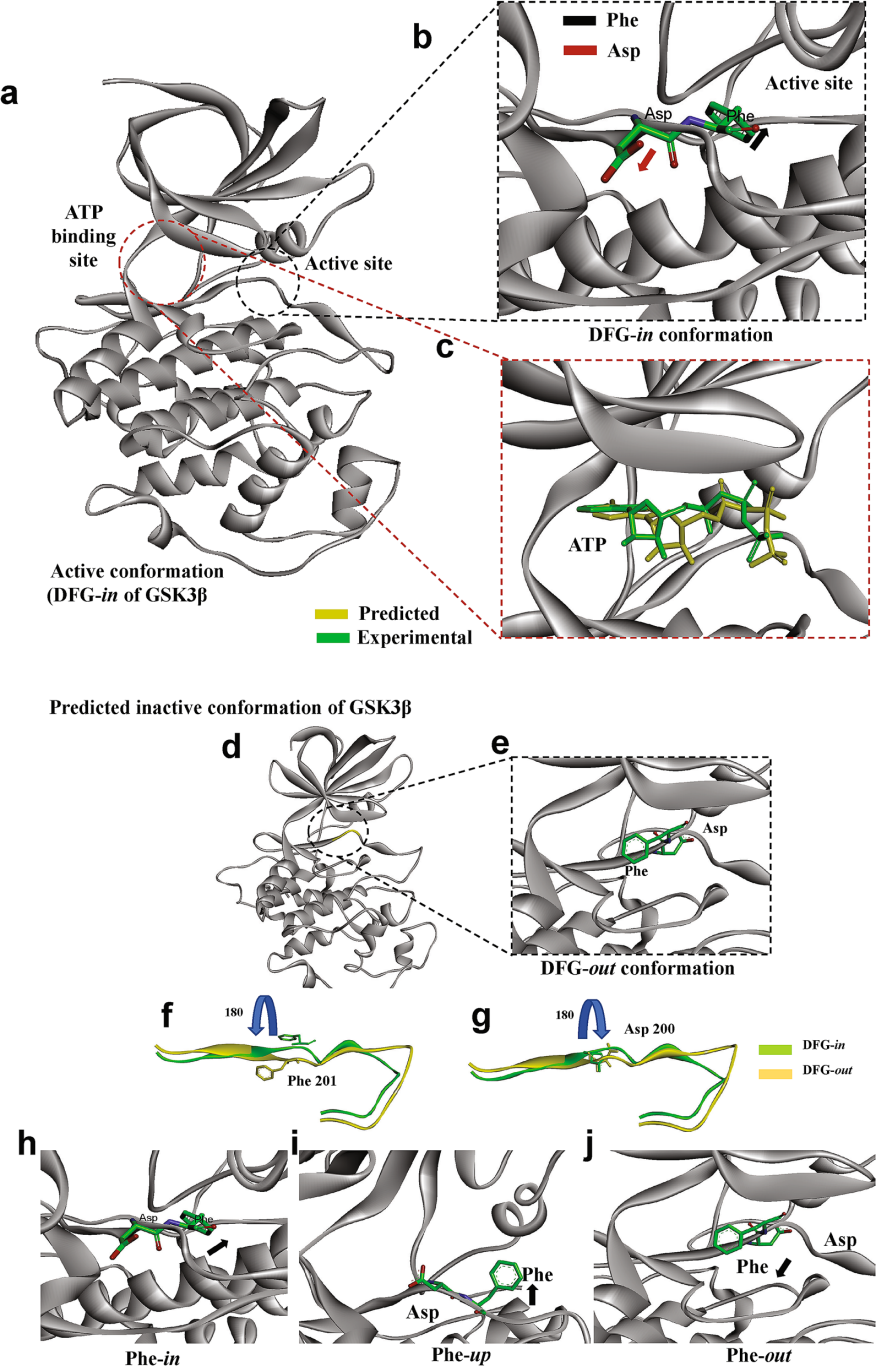
Active and inactive conformations of GSK3β. (**a**) Structure of active GSK3β, based on X-ray crystallography, showing the activation loop in the DFG-*in* conformation. (**b**) Stick representation of GSK3β residues Asp and Phe, showing the classical active (DFG-*in*) conformation, in which the Asp sidechain is rotated *out* and the Phe ring is turned *in* with respect to the active site. (**c**) Protein–ligand docking depicts ATP binding within its GSK3β binding site as predicted (yellow ATP structure) in close agreement with experimental data (green ATP). (**d, e**) Ribbon model of GSK3β inactive (DFG-*out*) conformation, in which Phe201 and Asp200 have rotated (flipped) their orientations relative to the active state. (**f,g**) Detail from (**e**), contrasting ribbon models of Phe201 and Asp200 in their active (green) vs. inactive (yellow) orientations, based on metadynamic simulation. (**h– j**) During metadynamic simulation, the transition of Phe201 from *in* to *out* conformation passes through an intermediate *up* orientation. Molecular structure depictions were created using the BIOVIA Discovery Studio Visualizer 2017 (Dassault Systemes; https://discover.3ds.com/discovery-studio-visualizer-download).

To model the inactive conformation of GSK3β, we began by considering its active conformation. Since the experimentally derived crystal structure of GSK3β omits several loops, we filled these gaps by template modeling (using the template PDB-ID: *1iep*) followed by loop refinement (Fig. 1a). The resulting structure of the activation loop predicts an outward orientation of aspartic acid D200, away from the ATP binding site, coupled to inward orientation of phenylalanine F201, facing the ATP pocket and the *N*-terminal αC-helix, i.e., the classical DFG-*in* conformation of active kinases (Fig. 1b). In order to evaluate the model that we generated and the computational parameters employed, we performed virtual docking of ATP with the modeled active conformation of GSK3β. Results obtained for binding of ATP to this predicted active conformation agree well with the experimentally determined active conformation of GSK3β (PDB-ID *1i09*) depicted in Fig. 1c.

We next attempted to model the inactive conformation of GSK3β by enhanced-sampling metadynamic simulation of the active conformation (see “Methods” for detailed procedures). In brief, the active conformation of GSK3β (Fig. 1a) was immersed in an orthorhombic box containing water plus salt ions (Na^+^, Cl^−^). The distance between the centers of mass of Phe200 and Ser168 was defined as a collective variable (CV) for metadynamic calculations. Simulation, conducted for 100 ns after equilibration, indicated a transition after ~ 18 ns involving both Phe and Asp in the DFG loop (Fig. 1d–e). At 10–25 ns, the DFG active conformation converted to the inactive conformation, i.e., Phe201 flipped almost 180° to face outwards (Fig. 1f), while Asp200 rotated inward 180° (Fig. 1g). Structural-alignment comparisons of this predicted DFG-inactive conformation demonstrated close agreement with the experimentally determined inactive conformations of other kinases, including ABL and AKT (Supplementary Fig. 1). Interestingly, our metadynamic simulation predicting the DFG-flip in GSK3β adopted an intermediate “*up*” conformation of Phe201 during transition to the *out* state (Fig. 1h–j; Supplementary Video S1). This transient *up* conformation was previously reported for several kinases, including the Aurora-A and Aurora-B kinases^11,21^.

In order to assess the reliability of the metadynamics approach as a means to predict the DFG-*out* conformation from the DFG-*in* structure, we used c-Abl kinase as a positive control. Since both the DFG-*in* (active) and DFG-*out* (inactive) conformations of c-Abl kinase have been resolved experimentally through X-ray crystallography, we began with the c-Abl kinase DFG-*in* crystal structure (PDBid: 3KF4) and repeated the identical steps used for GSK3β, to ask whether the predicted DFG-*out* conformation agreed with that empirically established crystal structure. As previously, the distance between the Phe381 and Ala350 centers of mass was defined as a collective variable (CV) for metadynamic calculations. Metadynamic simulation commencing with the c-Abl kinase DFG-*in* conformation predicts that a DFG-flip will occur at ~ 10–20 ns (Supplementary Fig. 2a–c), giving rise to a predicted DFG-*out* conformation that agrees remarkably well with the experimentally determined DFG-*out* structure of c-Abl (PDBid: 3KFA; Supplementary Fig. 2d).

### GSK3β transition to the inactive conformation creates a new hydrophobic/allosteric pocket

**Figure 2:**
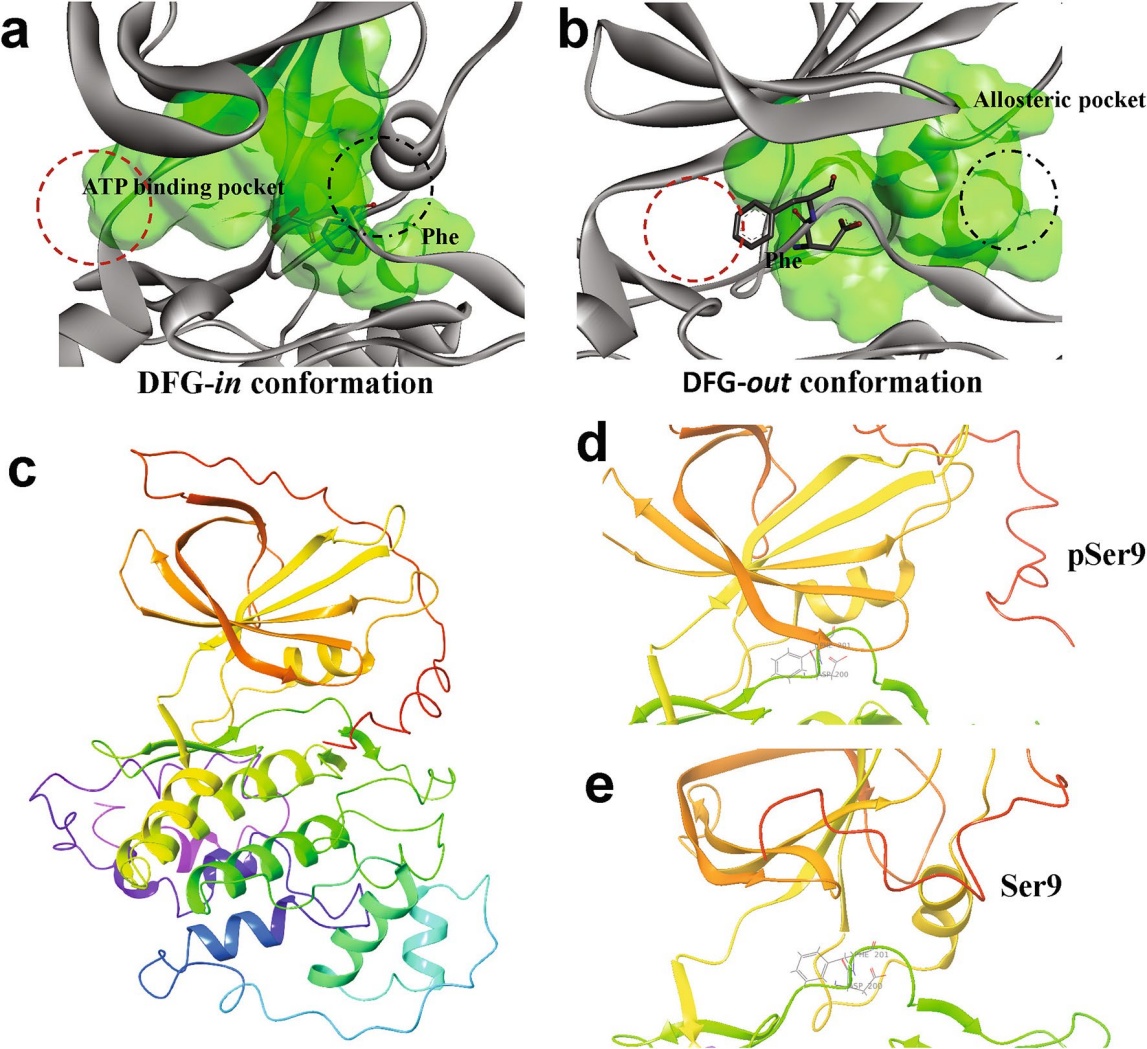
Modelled inactive (DFG-*out*) conformation of GSK3β shows structural changes near the active site. (**a**) Molecular surface representation (green) of predicted ATP-binding pocket when GSK3β is in the active conformation (DFG-*in*). (**b**) GSK3β spontaneously flips the DFG motif to the *out* (inactive) conformation during metadynamic simulation. The ATP-binding pocket is marked by a red dashed circle, whereas the allosteric hydrophobic pocket is designated by a black dashed circle. (**c–e**) Structural representations of modelled GSK3β (residues 1–384) in the inactive DFG-*out* conformation, with Serine9 either phosphorylated (**d**) or unphosphorylated (**e**)**.** Molecular structure representations were created using the BIOVIA Discovery Studio Visualizer 2017 (Dassault Systemes; https://discover.3ds.com/discovery-studio-visualizer-download), and Schrödinger Maestro 11.4 (https://www.schrodinger.com/).

Most kinases, in the inactive conformation, form a hydrophobic binding site adjacent to the ATP-binding pocket, termed the allosteric site^11–13^. Rotation of Asp and Phe in the DFG motif opens up a hydrophobic space between the *N*-terminal and C-terminal lobes^9^. Type-II kinase inhibitors, such as imatinib, generally target the inactive conformation of kinases, by interacting with the hydrophobic binding site (allosteric site) formed after DFG flip^9,13^. In order to ascertain whether our predicted inactive conformation incorporated this hydrophobic binding site, we modeled possible binding cavities in both the active and predicted inactive conformations. Results show a large cavity at the ATP-binding pocket in the active conformation of GSK3β (Fig. 2a, red dashed circle), which is absent in the predicted inactive conformation (Fig. 2b; red dashed circle). At the same time, the transition from active to inactive structures created a new hydrophobic area immediately adjacent to the ATP-binding pocket (Fig. 2b; black dashed circle), not seen in the active conformation (Fig. 2a; black dashed circle). These results lend indirect support for the DFG-flip predicted by metadynamic simulation, and are consistent with this being the true inactive conformation of GSK3β.

### Unphosphorylated Serine 9 delays the DFG flip (inactivation) in GSK3β simulations

Ser9 plays a critical role in GSK3β activity; its phosphorylation reduces the kinase activity of GSK3β, thereby suppressing phosphorylation of its downstream targets. However, the role of Ser9 phosphorylation in the DFG flip remains to be elucidated, in large part because most available experimental models of GSK3β omit the first 35 N-terminal residues. Therefore, using ab initio and fold-recognition protocols, we modeled the complete GSK3β structure (hereafter referred to as “full-length GSK3β”) including the *N*-terminal 35 residues (Fig. 2c). Structural analysis indicates that this modeled GSK3β assumes the active DFG-*in* conformation, and the position of the critical Ser9 in our full-length structural model agrees with the structure reported by Stamos et al.^22^.

We conducted a molecular-dynamic simulation of full-length GSK3β structural variation in the active conformation, using metadynamic simulation with the parameters noted above, including the same collective variable (CV) used previously to maintain consistency. In parallel, we phosphorylated full-length GSK3β at Ser9 (denoted as pSer9) using the Vienna PTM server (https://vienna-ptm.univie.ac.at/), and performed molecular simulation by the Metadynamics technique (see “Methods”)^11^. Both unphosphorylated and phosphorylated GSK3β models underwent a structural transition in the DFG region (Fig. 2d–e), from active to inactive conformation. However, this transition was delayed for full-length GSK3β with unphosphorylated Ser9, by ~ 40 ns relative to either phosphorylated full-length GSK3β (pSer9; Supplementary Fig. 3a–b) or GSK3β lacking the *N*-terminal 35 residues (18‒25 ns). Trajectory analysis revealed high instability (structural fluctuation) of the activation loop in unphosphorylated GSK3β, which was stabilized in pSer9-GSK3β (Supplementary Fig. 3a–b). The allosteric pocket formed during DFG transition includes a much smaller volume when GSK3β is unphosphorylated, relative to full-length GSK3β containing pSer9 (Supplementary Fig. 3c–e).

### TDZD-8 protects against aggregation in neurodegenerative models

**Figure 3:**
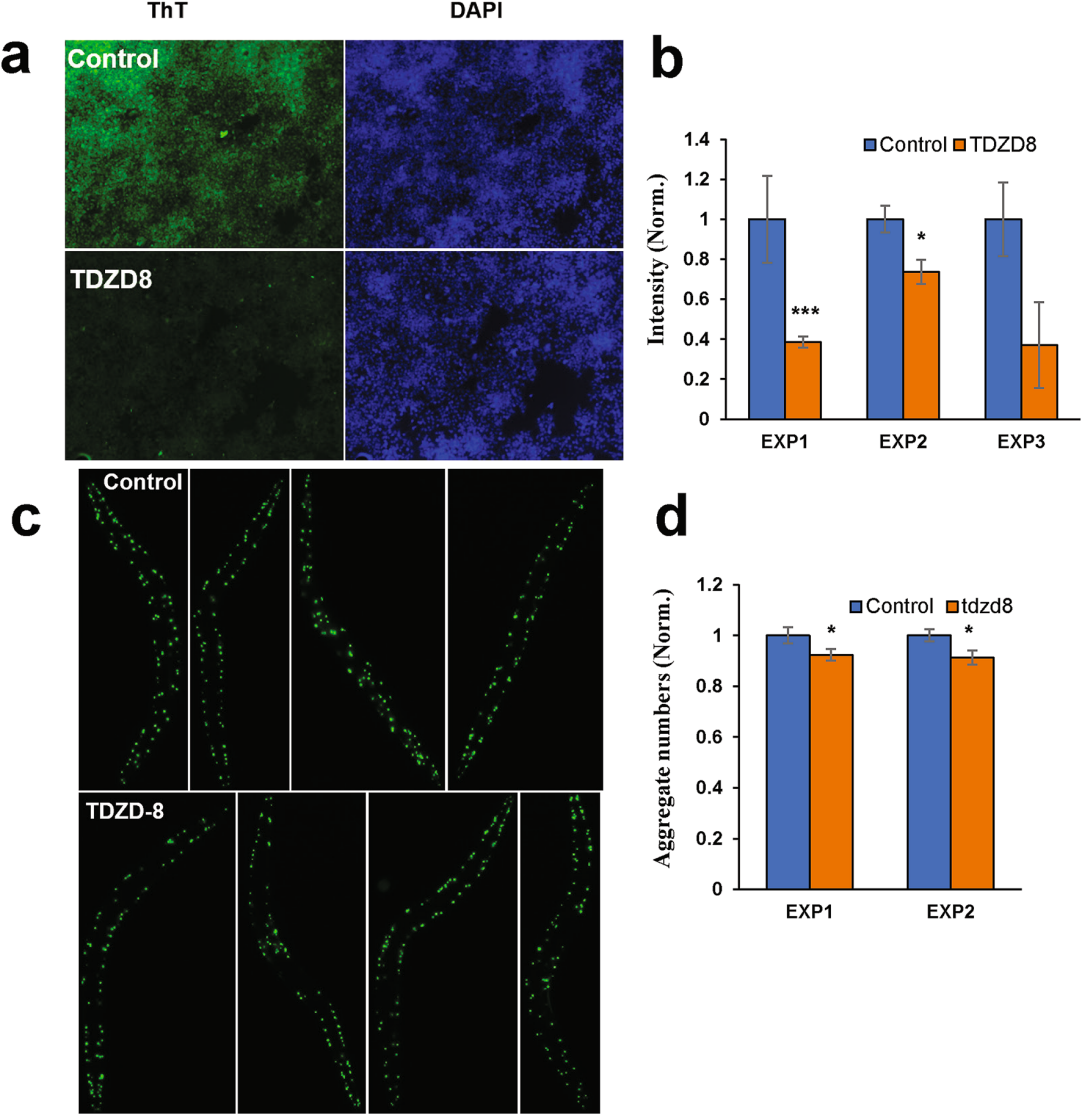
TDZD-8 treatment protects against amyloid-like aggregation in several model systems. (**a**) Thioflavin-T stains amyloid-like aggregates in HEK-tau cells, shown as controls (top panels) or after 48 h exposure to 5-μM TDZD-8 (lower panels). (**b**) Normalized fluorescence intensities are shown for three independent experiments, in 2 of which treatment differed from control by 1-tailed *t*-test: *P < 0.05; ***P < 0.001. (**c**) Images of *C. elegans* adults (strain AM141, *unc-54p::Q40 ::yfp*) from the control group (top panel) or the drug-treated group (50-µM TDZD-8) at day-5 post-hatch. (**d**) Normalized aggregate counts per worm, ± SEM, were calculated and plotted for 10–12 worms per group in two independent experiments. Significance, calculated by 1-tailed *t*-tests: *P < 0.05.

Previous studies have shown that TDZD-8 inhibits GSK3β and thus protects against tau hyperphosphorylation^23^, and that other small-molecule inhibitors of GSK3β protect against Aβ_42_ aggregation to form β-amyloid^24,25^. We assessed the ability of TDZD-8 to protect human cells against tau aggregation in HEK-tau embryonic kidney cells expressing a MAPTAU transgene. Thioflavin-T-positive AD-like aggregates were significantly reduced in HEK-tau cells by 5-µM TDZD-8 (Fig. 3a,b), consistent with GSK3β playing a critical role in AD-like aggregation.

In addition to this cell model of AD neuropathy, we also assessed a nematode model of Huntington’s disease (*Caenorhabditis elegans* strain AM141), expressing an array of 40 glutamines fused to Yellow Fluorescent Protein (Q40::YFP). Interestingly, even without dose optimization, TDZD-8 treatment substantially attenuated aggregate accumulation in this model, suggesting the possibility that GSK3β or its downstream targets may also be involved in Huntington’s and/or other polyglutamine-array-expansion diseases (Fig. 3c,d).

### TDZD-8 binds to the inactive conformation of GSK3β

Having now modelled full-length GSK3β in both the active and inactive conformations, we simulated its interaction with TDZD-8 in order to clarify the mode of drug binding. We began with the full-length active conformation of GSK3β, and used Glide to perform protein–ligand targeted docking within the previously proposed TDZD-8-binding region of GSK3β^19^. TDZD-8 was not a good fit to the proposed region (the area around the allosteric/hydrophobic pocket) of the GSK3β active conformation (Supplementary Fig. 4), presumably because this (active) conformation does not form a stable hydrophobic pocket (Fig. 2a). To determine whether TDZD-8 binds to any region of the GSK3β active conformation beyond the proposed pocket, we employed AutoDock-Vina to perform unbiased docking, wherein the entire active conformation of full-length GSK3β is enclosed in the docking box^26^. None of the predicted docking poses resulted in TDZD-8 binding to the proposed allosteric/hydrophobic site of GSK3β in the active conformation (data not shown). Although unbiased docking indicates modest TDZD-8 affinity for the ATP-binding pocket of the active-conformation GSK3β (Supplementary Fig. 4), calculation of the solvent-based binding free energy (by the MMGBSA approach^27,28^) predicted that the binding affinity of TDZD-8 for this pocket would be less than half that of ATP (Supplementary Fig. 4d). This indicates that the drug could not compete effectively for the ATP binding site, consistent with previous biochemical evidence that TDZD-8 is an ATP-noncompetitive inhibitor^29^.

**Figure 4:**
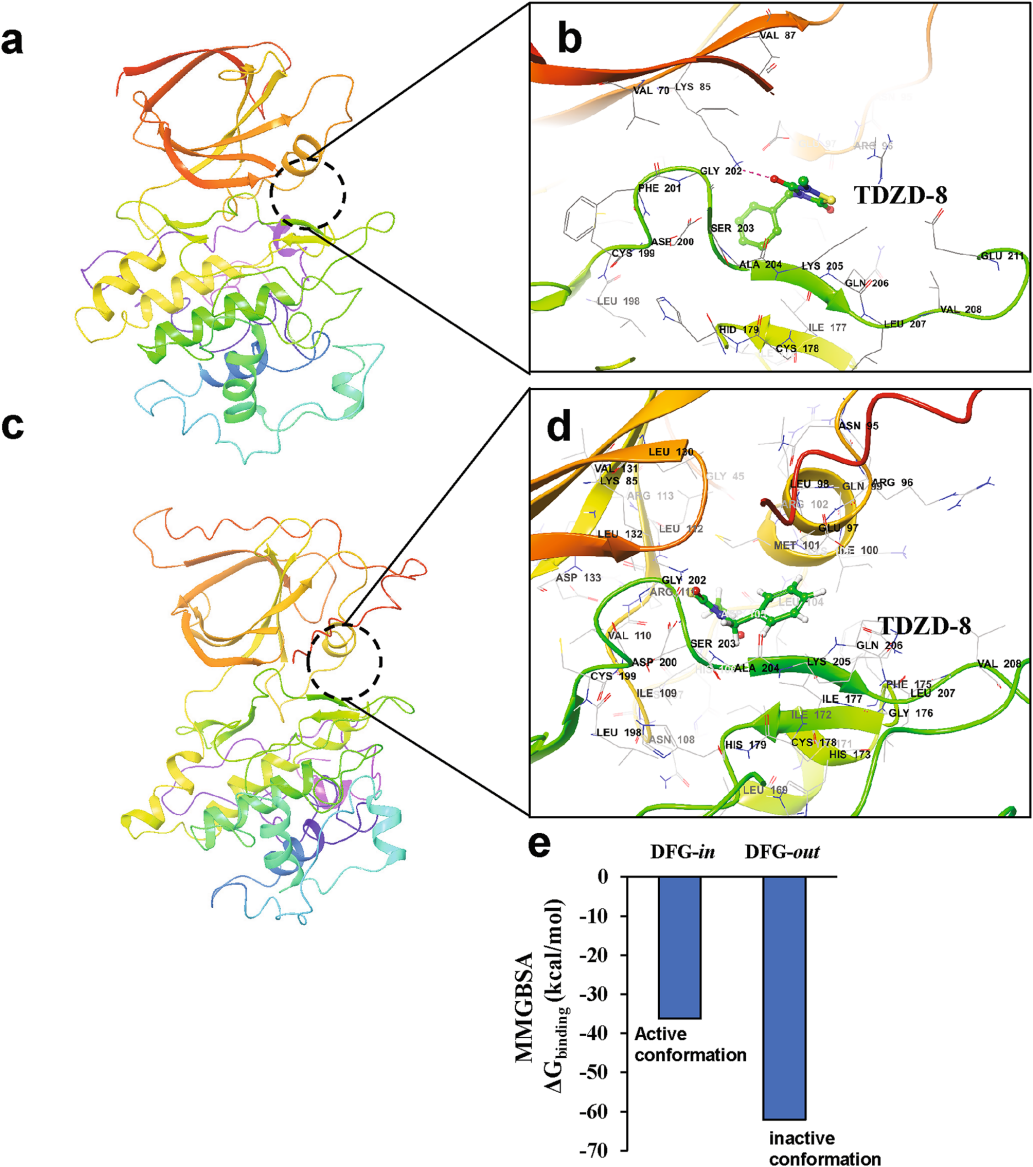

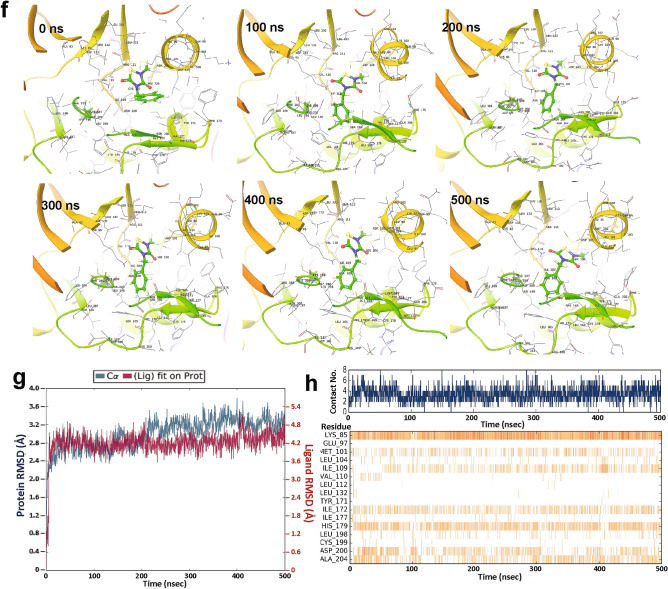
TDZD-8 binds to the allosteric hydrophobic pocket of GSK3β. (**a,b**) Glide docking pose of TDZD-8 binding to the GSK3β allosteric hydrophobic pocket in the modelled inactive DFG-*out* conformation. (**c,d**) TDZD-8 docked pose with modelled full-length GSK3β-pSer9 in the inactive DFG-*out* conformation. (**e**) MMGBSA-based ΔG_binding_ (Gibbs free energy of binding) calculated for TDZD-8 with GSK3β in both active (DFG-*in*) and inactive (DFG-*out*) conformations. (**f**) Snapshots from 0.5-µs simulation of full-length GSK3β (pSer9)-TDZD-8 complex taken at 100-ns intervals. (**g**) Root Mean Square Deviation of protein–ligand complex during a 0.5-µs simulation; the blue and red tracings represent RMSD for GSK3β and TDZD-8, respectively. (**h**) Total number of contacts/interactions between TDZD-8 and GSK3β vs. time (top panel); stabilities of 16 individual interactions during the simulation (lower panel). Molecular structures are depicted with Schrödinger Maestro 11.4 (https://www.schrodinger.com/).

Since the proposed hydrophobic pocket in GSK3β is only present when the kinase is in the inactive conformation, we subjected that model to unbiased docking followed by target-specific docking of TDZD-8, as described above. In both procedures, TDZD-8 showed stable binding to the hydrophobic pocket adjacent to the ATP-binding site (Fig. 4a–d). Notably, TDZD-8 binding to this pocket is identical for the predicted inactive conformations of both full-length GSK3β and its truncated form, which lacks the *N*-terminal 35 residues (Fig. 4a–d). Analysis of the contact residues agrees with the binding mode proposed previously for TDZD-8 interaction with GSK3β^19^. We calculated the solvent-based binding free energy (ΔG_binding_) of TDZD-8 targeted to the hydrophobic/allosteric pocket residues, using MMGBSA (molecular mechanics under the generalized Born model with solvent accessibility). For TDZD-8 docking to the inactive conformation of GSK3β, ΔG_binding_ was nearly twice that for the active conformation (Fig. 4e).

### Atomistic MD simulation shows stable TDZD-8 binding at the GSK3β hydrophobic pocket

In order to evaluate the stability of TDZD-8 binding to the hydrophobic pocket of GSK3β in its inactive conformation, we conducted a 0.5-µs, fully solvated atomistic molecular-dynamic simulation. The results indicate stable binding of TDZD-8 throughout the simulation. Snapshots taken at 100-ns intervals show continuous TDZD-8 occupation of the allosteric hydrophobic pocket between helix αC of the *N*-terminal lobe and the catalytic domain (Fig. 4f). RMSD calculations indicate that GSK3β did not undergo major structural changes during the simulation (Fig. 4g; RMSD range is < 1 Å for both the protein and ligand). RMSD trajectories for TDZD-8 complexed with GSK3β indicate stable drug binding in the hydrophobic pocket of GSK3β (Fig. 4g; dark red tracing). Since the number of amino acid-ligand contacts is crucial to the stability of a drug-protein complex, we monitored the interactions between TDZD-8 and GSK3β residues over time. TDZD-8 interacts throughout most of the simulation with 2‒6 GSK3β residues, averaging ~ 3 (Fig. 4h). The most consistent contact residues were Lys85 and Asp200/ Ala204.

### Computational docking and pharmacophore analysis of known inhibitors of GSK3β in the inactive conformation

**Figure 5:**
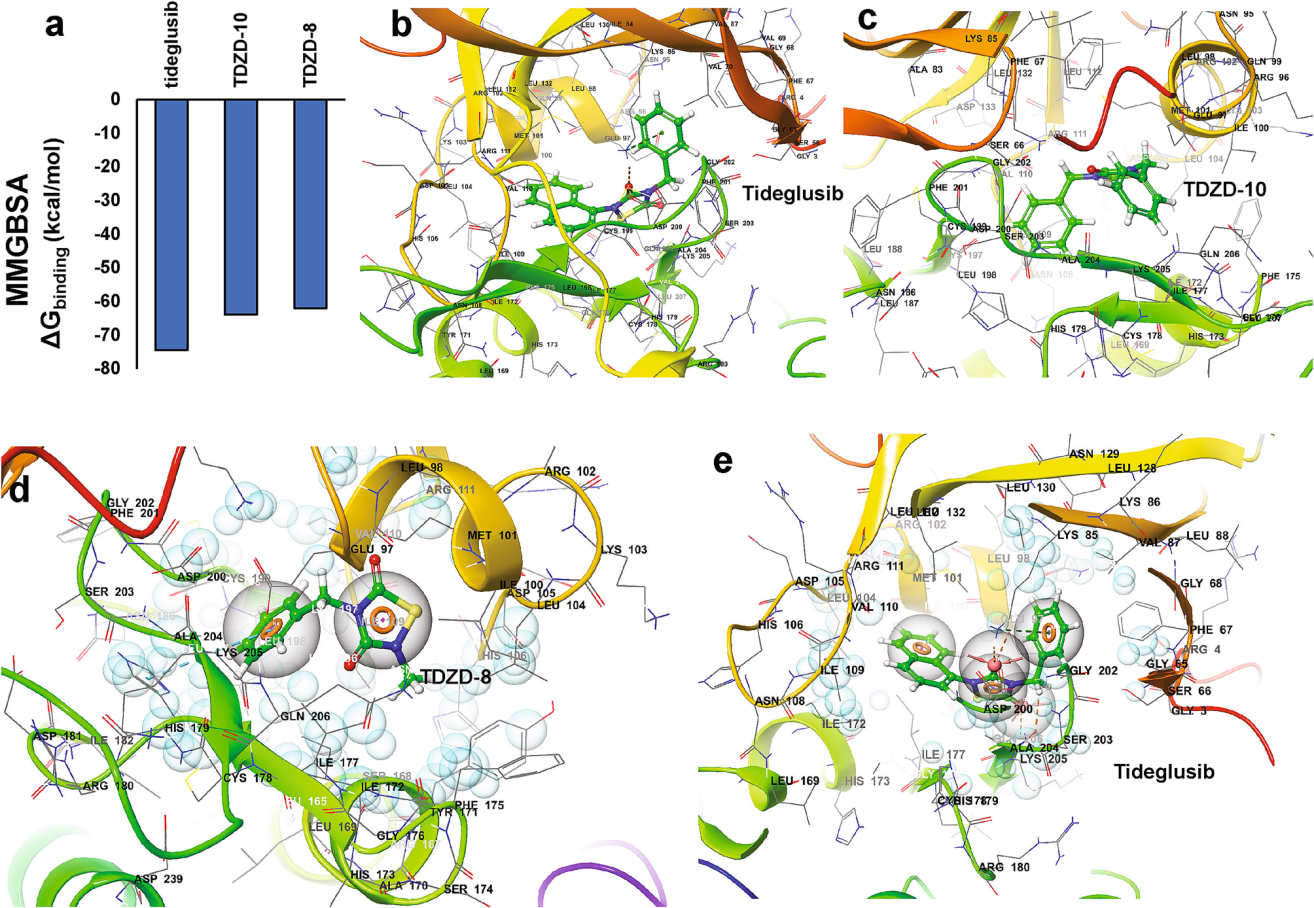
Binding modes of TDZD analogs with GSK3β inactive conformation. (**a**) ΔG_binding_ (Gibbs free energy of binding), calculated by MMGBSA for 3 potent TDZD analogs, with GSK3β in the inactive conformation. (**b,c**) GSK3β docking poses with tideglusib and TDZD-10, indicate binding similar to that of TDZD-8 to the proposed allosteric hydrophobic pocket. (**d,e**) Pharmacophore modeling, based on predicted protein–ligand complexes, predicts key features involved in TDZD-8 and tideglusib binding. Molecular structures were created with Schrödinger Maestro 11.4 (https://www.schrodinger.com/).

Since the discovery of TDZD-8, many inhibitors, including second-generation TDZD-8 analogs (e.g. TDZD-10 and Tideglusib), have been proposed and some of these were shown to be effective ATP-noncompetitive inhibitors of GSK3β^20,30,31^. However, the binding modes of these compounds were inadequately determined due to the lack of an empirically derived inactive conformation of GSK3β. In order to test the reliability of our predicted inactive conformation of GSK3β, we modeled the binding modes of previously known ATP-noncompetitive inhibitors of GSK3β against our predicted inactive conformation of GSK3β. These other known TDZD analogs that are ATP-noncompetitive inhibitors of GSK3β are predicted to bind at the hydrophobic pocket in the inactive conformation, with very high binding affinity (Fig. 5a). We tested Tideglusib, the best of the ATP-noncompetitive (TDZD) inhibitors of GSK3β, and showed that it binds at the interface between the *N*-terminal helix and the activation loop (i.e., in the hydrophobic/allosteric pocket), extending towards the active site (Fig. 5b). TDZD-10 (PubChemID:6539140; a.k.a. compound-45)^31^ also fits well within the GSK3β hydrophobic pocket (Fig. 5c).

We next attempted to predict and model the common pharmacophore of TDZDs based on the protein–ligand complex we had modeled. Using Phase pharmacophore modeling from the Schrödinger suite, we predicted the pharmacophore of TDZD-8 when bound to GSK3β as a protein–ligand complex. The modeling algorithm proposed three key pharmacophore features (active components for target binding) for TDZD-8 binding (Fig. 5d): the TDZD ring (R3 in Fig. 5d) that participates in π-alkyl interactions, the carbonyl group attached to the TDZD ring (A2 in Fig. 5d) that forms an H-bond with Lys85, and the benzene ring (R4 in Fig. 5d) that participates in π-alkyl interactions. Pharmacophore modeling for Tideglusib, which targets the same reported critical residues in the activation loop (e.g. Lys205 and Asp200/Ala204), adds a naphthalene ring (R5) not present in TDZD-8, improving its binding affinity to GSK3β by adding π-alkyl bonds with Ile177 and Met101 (Fig. 5e). A comparison of the predicted pharmacophore features of the two TDZD analogs (TDZD-8, and Tideglusib) shows that the TDZD ring (R3) and carbonyl moiety (A2) remain the key common pharmacophore features of these TDZD analogs.

## Discussion

In this study, we modelled the inactive “DFG-*out*” conformation of GSK3β using metadynamic simulation (schematic representation; Supplementary Fig. 5). Previous studies showed that inhibition of GSK3β by a small-molecule drug blocks phosphorylation of tau and prevents its hyperphosphorylation—a step that initiates the formation of neurofibrillary tangles (NFT), the hallmark diagnostic feature of Alzheimer’s disease^4,19,20,32^. Many competitive inhibitors of GSK3β have been developed to target the catalytic (ATP-binding) site of the kinase in its active form/conformation, and are thus obliged to compete with ATP for binding^33,34^. Although GSK3β is often considered to be constitutively active, there is strong evidence that Ser9 phosphorylation induces kinase inactivation and inhibits substrate binding^22,35,36^, perhaps by favoring the DFG-*out* (inactive) conformation. Considering that GSK3β has the key DFG regulatory motif in its activation loop, in common with other Ser/Thr kinases, we hypothesized that Ser9 phosphorylation is likely to favor a flip to the inactive conformation, thereby inhibiting substrate binding.

Small molecules have been identified that inhibit the activity of a target kinase but do not compete with ATP for binding (i.e., they are non-competitive)^19^. In general, ATP-noncompetitive inhibitors are known to target an allosteric/hydrophobic pocket, observed in the inactive forms of several kinases^9,37^. Such inhibitors have two advantages over ATP-competitive inhibitors: (i) they often show greater target selectivity, since they do not bind to the active form of the kinase, evading the ATP-binding pocket that is largely conserved across the kinases; and (ii) they do not compete for binding with ATP at the ATP binding site on the active form of the kinase. ATP is an abundant molecule with intracellular concentrations of 0.1‒10 mM^38^, which has very high affinity for the ATP-binding sites of kinases in their active conformations.

TDZD-8 is an example of an ATP-noncompetitive inhibitor of GSK3β, and was proposed to target an allosteric hydrophobic pocket on the kinase^19^. TDZD analogs show relatively high specificity for GSK3β, resulting in potent inhibition and consequent amelioration of specific neuropathologies^19,23^. Several analogs of TDZD exhibit enhanced inhibitory activity against GSK3β relative to the parent compound^20,31^. Because GSK3β is widely considered to be constitutively active, it was long believed that TDZD analogs target an allosteric hydrophobic site in its active conformation. However, structural details of GSK3β’s interactions with TDZD-8 and its more potent analogs have eluded experimentally resolution. Our computational analysis predicts that TDZD-8 would not show affinity for the active form of GSK3β, which appears to lack any allosteric hydrophobic pocket. This suggests that TDZD might instead bind to a hydrophobic pocket in the inactive form of the kinase, similar to Imatinib, another ATP-noncompetitive inhibitor that targets the inactive form of Abl kinase^9^. Prior to the current study, no experimentally determined structure had been reported for the inactive conformation of GSK3β, and very little was known about the binding modes or pharmacophores of TDZD-8 and its analogs. This gap in our knowledge has impeded the creation of next-generation TDZD analogs.

Atomistic metadynamic simulation has been a useful tool for predicting large conformational changes in the three-dimensional structures of proteins. We have applied metadynamic modeling to derive the inactive conformation of GSK3β from its known active conformation (based on X-ray crystallographic data^10^). Since metadynamics is an enhanced sampling method, it is able to predict conformational changes on a nanosecond scale, which is more than sufficient to predict the DFG-flip we observed at 18‒25 ns. The predicted DFG-flip agrees with the available inactive conformations of other known kinases, including the Abl kinase structure empirically derived from X-ray data. Previous studies of other kinases have documented that the DFG-flip creates an allosteric pocket adjacent to the ATP binding site, consistent with cavities appearing in our predicted GSK3β inactive conformation, but absent from our starting model representing the active conformation. Another interesting feature of our predicted model is that the DFG-flip from an active to inactive state reduces the volume of the ATP-binding pocket. This corroborates previous evidence that ATP binding is attenuated upon kinase inactivation. At the same time, since non-competitive inhibitors are often stronger inhibitors of kinase activity^9^, novel non-competitive inhibitors can be identified by structure-based drug discovery using our predicted model.

Since phosphorylation of Ser9 plays a critical role in the activity of GSK3β, and the previously available experimental structures lack the *N*-terminal 35 residues, we first derived a full-length model of GSK3β structure in its active conformation. Metadynamic simulation showed that DFG-flip occurs spontaneously within the first few nanoseconds of simulation if Ser9 is phosphorylated. Interestingly, inactivation via DFG-flip was delayed when Ser9 was not phosphorylated, and the inactive conformation was unstable. This supports the hypothesis that Ser9 phosphorylation triggers inactivation of GSK3β. Apart from inhibiting substrate binding, Ser9 phosphorylation could also favor the DFG-flip to an inactive GSK3β conformation.

Computational docking of TDZD-8 with active-conformation GSK3β is not consistent with the previously proposed mode of TDZD-8 binding^19^. I.e ., the proposed binding residues adjacent to the catalytic site cannot be reconciled with our docking model due to the absence of an allosteric hydrophobic pocket. Instead, the model predicts TDZD-8 binding to the proposed allosteric pocket of the inactive conformer of GSK3β. The predicted binding residues agree with previous reports for TDZD-8 binding, and thus support the predicted TDZD-8 binding to GSK3β in its inactive conformation. Additional support comes from the stronger affinity (more negative ΔG_binding_) of TDZD-8 for GSK3β in the inactive rather than the active conformation. Molecular docking of other known GSK3β ATP-noncompetitive inhibitors predicts their modes of binding to the GSK3β inactive conformation. Interestingly, docking results predict that tideglusib, a potent and selective ATP-noncompetitive inhibitor of the thiadiazolidinone (TDZD) class of small molecules, should interact at the allosteric pocket of the GSK3β inactive conformation with high binding affinity relative to other known ATP-noncompetitive inhibitors, including palinurin and TDZD-10.

Interestingly, Tideglusib is known to inhibit GSK3β irreversibly^20^. This led to speculation that Tideglusib might form a covalent bond with Cys199 of GSK3β, but biochemical studies found no evidence of covalent linkage to Tideglusib at that residue^20^. Now, with the benefit of a structural model for the drug-protein complex, we have re-examined this putative mechanism of irreversible Tidelusib binding. Our structural model of GSK3β (inactive) binding by Tideglusib shows that the TDZD ring of Tideglusib does not face toward Cys199 (Fig. 5), making a covalent bond there unlikely. However, both modeling and docking studies of the Tideglusib-GSK3β (DFG-*out*) complex show the TDZD ring of Tideglusib in close proximity to Cys178 of GSKβ, in an orientation that could allow covalent-bond formation. Further biochemical studies will be required to test this possibility. Lead optimization of TDZD-8 analogs, based on a more compelling predicted binding mode, would provide significant assistance in designing more potent and selective inhibitors of GSKβ (manuscript in preparation). Results of the current study substantiate the important role of the GSK3β inactive conformation in the mechanism of non-competitive inhibition by TDZD-8 and its structural analogs.

## Methods

### Structure modeling and system building for MD runs

The active (“DFG-*in*”) conformation of GSK3β was retrieved from the Protein Data Bank (PDB-ID ***1I09***), from which we removed all crystal-associated water molecules and other heteroatoms associated with the structure. Prior to docking and simulation studies, GSK3β structure was preprocessed by adding missing hydrogens, side-chains, and loops, using the Protein Preparation Wizard within the Maestro Prime module (Schrodinger, Inc.). This preprocessed GSK3β active conformation was used for all further computational modeling studies in this manuscript. The active and inactive structure of c-Abl kinase were retrieved from Protein Data Bank (PDB-IDs ***3KF4*** and ***3KFA*** respectively). Before simulations, the 3KF4 (active) structure of c-Abl kinase was loop refined and missing atoms/residues were added using Modeller V9.19.

### System building and metadynamic simulations

The active conformation of GSK3β, prepared as above, was centered in an orthorhombic box positioned so that all box edges were 10-Å removed from the protein. This simulation box was solvated with Simple Point Charge (SPC) water and neutralized with Na^+^, Cl^–^ counterions. NaCl at 0.15 M was then added to establish physiological salt concentration in the simulation box. The simulation system was then well equilibrated at 300^o^K using the NVT protocol with a Nose–Hoover chain thermostat. This was followed by another equilibration phase before the actual Molecular Dynamics (MD) run using the NPT protocol. The actual MD run followed a metadynamic simulation protocol as described previously^11,39^. Briefly, for the metadynamics run, collective variables (CVs) play crucial roles^40^. For our simulations, the distance between Phe 201 and Ser 168 in the GSK3β active structure (1I09) was designated as a CV with Gaussian width of 0.05 Å. Simulations were conducted in GPU accelerated Desmond v2018.1 running on an NVIDIA Quadro P5000 cluster system developed in-house. Metadynamic simulations were conducted at a temperature of 300^o^K and pressure of 1.0 bar, with RESPA integrator.

### Atomistic molecular-dynamic simulations

To analyze the stability of the protein–ligand complex, regular atomistic molecular-dynamic simulations were conducted using GPU accelerated Desmond v2018.1 with previously employed parameters ^26,41^. Briefly, the protein–ligand complex was preprocessed as above using the Protein Preparation Wizard, and immersed in a orthorhombic box containing SPC water molecules. The simulation box was neutralized using Na^+^, Cl^**–**^ as counterions with addition of 0.15-M NaCl as required. The well-equilibrated system was then simulated using the RESPA integrator at a temperature of 300^o^K and pressure of 1 bar. Trajectories were analyzed using the Simulation Interaction Diagram Generator from Desmond-Maestro.

### Molecular docking

For unbiased docking, was we used Linux-based Autodock-Vina with the Raccoon interface. The docking grid box was was structured to cover the entire molecule, as described previously ^26,42^. Target-specific (i.e. site-directed) docking and pose prediction were performed using the Glide docking interface within Maestro 2017-2 Suite (Schrodinger) running on a commercial license. The docking grid box was generated using the Receptor Grid Generator Wizard by manually selecting residues from either the active site or from the allosteric hydrophobic pocket. Docking calculations were performed in standard precision mode with flexible ligand sampling. Results were visualized and analyzed using the Maestro Viewer and Discovery Studio Visualizer.

### MMGBSA calculation of binding energy

The docking pose from the Glide docking provided the initial input to calculate the solvent-based free energy of binding (ΔG_binding_). Binding free energy of the input ligand was estimated by the MM-GBSA method (Molecular Mechanics using the Generalized Born model and Solvent Accessibility), as implemented in the Prime module of Schrodinger Suite.

### Pharmacophore modeling

The output pose from Glide docking was used to generate a proposed pharmacophore based on the protein–ligand complex. The Auto E-pharmacophore method from the Phase plugin (Schrodinger Suite) was employed with default rules to formulate pharmacophore hypotheses, which were then visualized using Maestro Viewer.

### Strains, maintenance, and visualization of aggregates

*C. elegans* strain AM141 [*unc-54p::Q40::yfp*] was obtained from the Caenorhabditis Genetics Center (CGC). This strain was maintained on a regular solid-agar nematode growth medium at 20 °C, seeded with OP50 (*E. coli*) bacteria. TDZD-8 compound was synthesized in-house and reconstituted as a stock solution in DMSO, which was then diluted in PBS and an amount was added to the agar plate 1–3 days prior to use, calculated to bring it to the working concentration specified in the paper once evenly diffused. Eggs from the well-fed AM141 strain were seeded onto control and experiment plates, maintained at 20 °C. Both controls and TDZD-8-treated worms, at day 5 post-hatch (approximately day 3 of adulthood), were imaged on a Keyence BZ-810 fluorescence microscope. Q40::YFP aggregates from the captured images were identified, counted, and quantified using Fiji image processing within ImageJ.

### Cell culture, maintenance, and thioflavin-T fluorescence assay

Human embryonic kidney cells expressing human tau (HEK-tau) provided a model of human tauopathy, kindly provided by Dr. Brian Kraemer^43^. For regular maintenance, both cell types were plated at 250,000 cells per T-75 flask containing DMEM-F12 (1:1) supplemented with 10% v/v fetal bovine serum, and were maintained at 37 °C in a 5% CO_2_ atmosphere with 95% relative humidity, in a tissue-culture incubator (FisherBrand). For experiments with drug treatment cells were plated at 6000 cells per well in a 96-well tissue culture treated plate (Costar). Drug-treated cells and controls were stained with thioflavin-T as previously described^41,44,45^. Cells were fixed after 48-h drug exposure, and incubated in phosphate-buffered saline containing 0.1% (w/v) thioflavin-T dye, and 4′,6-diamidino-2-phenylindole (DAPI). After three successive 2-min washes, cells were imaged using a Keyence BZ-810 fluorescence microscope. After background subtraction at a rolling-ball radius of 50 (using Keyence image-analysis software), aggregate fluorescence was quantified from the images using Fiji (ImageJ).

### Statistical analyses

The significance of differences between groups were calculated using the Behrens-Fisher heteroscedastic *t* test, appropriate to small samples (*N* = 4) for which the variance is poorly defined or unequal. Single-tailed *t* tests are employed whenever the direction of change is known based on previous data; no Bonferroni correction has been applied, as only single endpoints were tested for intergroup significance.

## Supplementary information

Supplementary Information 1.

Supplementary Video 1.

## Data Availability

The predicted inactive conformation of GSK3β, presented here, can be retrieved from this site: https://data.mendeley.com/datasets/d69pzg3syh/1.
