# Supplementary material for: Structural modeling of GSK3β implicates the inactive (DFG-out) conformation as the target bound by TDZD analogs
Source: Sci Rep. 2020 Oct 27;10:18326. doi: 10.1038/s41598-020-75020-w (PMC7591898; doi:10.1038/s41598-020-75020-w)

Structural modeling of GSK3β implicates the inactive (DFG-*out*) conformation as the target bound by TDZD analogs

Meenakshisundaram Balasubramaniam[1,2*], Nirjal Mainali[3], Suresh Kuarm Bowroju[5], Paavan Atluri[4], Narsimha Reddy Penthala[5], Srinivas Ayyadevera[1,2], Peter A. Crooks[5], Robert J. Shmookler Reis[1,2*]

[1] McClellan Veterans Medical Ctr., Central Arkansas Veterans Healthcare Service, Little Rock AR 72205, USA.

[2] Dept. of Geriatrics, Reynolds Institute on Aging, University of Arkansas for Medical Sciences, Little Rock, AR 72205 USA.

[3] Bioinformatics Program, University of Arkansas for Medical Sciences, and University of Arkansas at Little Rock, Little Rock AR 72205 USA.

[4] Summer Research Internship Program, University of Arkansas for Medical Sciences, Little Rock, AR 72205 USA.

[5] Department of Pharmaceutical Sciences, College of Pharmacy, University of Arkansas for Medical Sciences, Little Rock, AR 72205.

[*] Correspondence to reisrobertj@uams.edu or mbalasubramaniam@uams.edu

Supplementary Material

Supplementary Figure 1: Comparison of modelled DFG-*out* loop region of inactive GSK3β with other known experimentally resolved DFG-*out* loops of three well-studied, inactive kinases: ABL, AKT, and MEK1. Molecular structure depictions were created using the BIOVIA Discovery Studio Visualizer 2017 (Dassault Systemes; https://discover.3ds.com/discovery-studio-visualizer-download).

Supplementary Figure 2: Metadynamic simulation of c-Abl kinase DFG-*out* conformation from the experimental DFG-*in* conformation (**3KF4**). (**a**) Structure of c-Abl kinase DFG-*in* conformation showing Phe382 facing "inward" and Asp381 facing "outward". (**b**) Metadynamic- simulated structure of predicted DFG-*out* conformation showing the DFG-flip, such that Phe382 faces "outward" and Asp381 faces "inward". (**c**) Root-mean-square deviation from initial starting conformation calculated for 30 ns metadynamic trajectories. (**d**) Structural alignment between experimental DFG-*out* (inactive) conformation of c-Abl with our DFG-*out* (inactive) conformation of c-Abl predicted by metadynamic simulation. Molecular structures were created with Schrödinger Maestro 11.4 (https://www.schrodinger.com/).

Supplementary Figure 3: Phosphorylation of Ser9 is predicted to alter GSK3β structure. **(a)** Root Mean Square deviation (RMSD) of GSK3β phosphorylated at Ser9 (pSer9), modeled for 50 ns; **(b)** RMSD of unphosphorylated GSK3β (Ser9). (**c – e**), Calculation of binding-pocket volume predict pronounced pocket expansion upon Serine9 phosphorylation. (**c**) Simulated DFG-*out* conformation with phosphorylated serine (pSer9) shows a much larger allosteric hydrophobic pocket than predicted in **(d),** the DFG-*out* conformation with unmodified Serine9. **(e)** The potential occupancy volume predicted for the allosteric hydrophobic pocket expands >4-fold when the GSK3β inactive conformation contains pSer9 (phosphorylated Serine 9). Molecular structure depictions were created using the BIOVIA Discovery Studio Visualizer 2017 (Dassault Systemes; https://discover.3ds.com/discovery-studio-visualizer-download).

Supplementary Figure 4: TDZD-8 could not serve as an effective ATP-competitive inhibitor of GSK3β in its active conformation. (**a**) The active conformation of GSK3β is shown, with an ATP-binding pocket (red dashed circle). (**b**) The accessible binding-pocket volume is shown in green. (**c**) TDZD-8 is shown in the ATP-binding pocket. (**d**) The absolute value of $\Delta G_{binding}$ (Gibbs Free Energy of binding) for ATP exceeds that for TDZD-8 by more than twofold. Molecular structure depictions were created using the BIOVIA Discovery Studio Visualizer 2017 (Dassault Systemes; https://discover.3ds.com/discovery-studio-visualizer-download).

Supplementary Figure 5: Schematic representation of computational strategy used to predict the DFG-*out* conformation of GSK3β.

Supplementary Video: This animation, covering the first 25 nsec of the simulation in Supplementary Figure 2b, shows the predicted transition from DFG-*in* to DFG-*out* based on metadynamic simulation trajectories. Stick structures represent Phe201 (orange) and Asp200 (white). Video animation was generated using VMD 1.9.3 (https://www.ks.uiuc.edu/Research/vmd/).

Balasubramaniam et al., Supplementary Figure 1

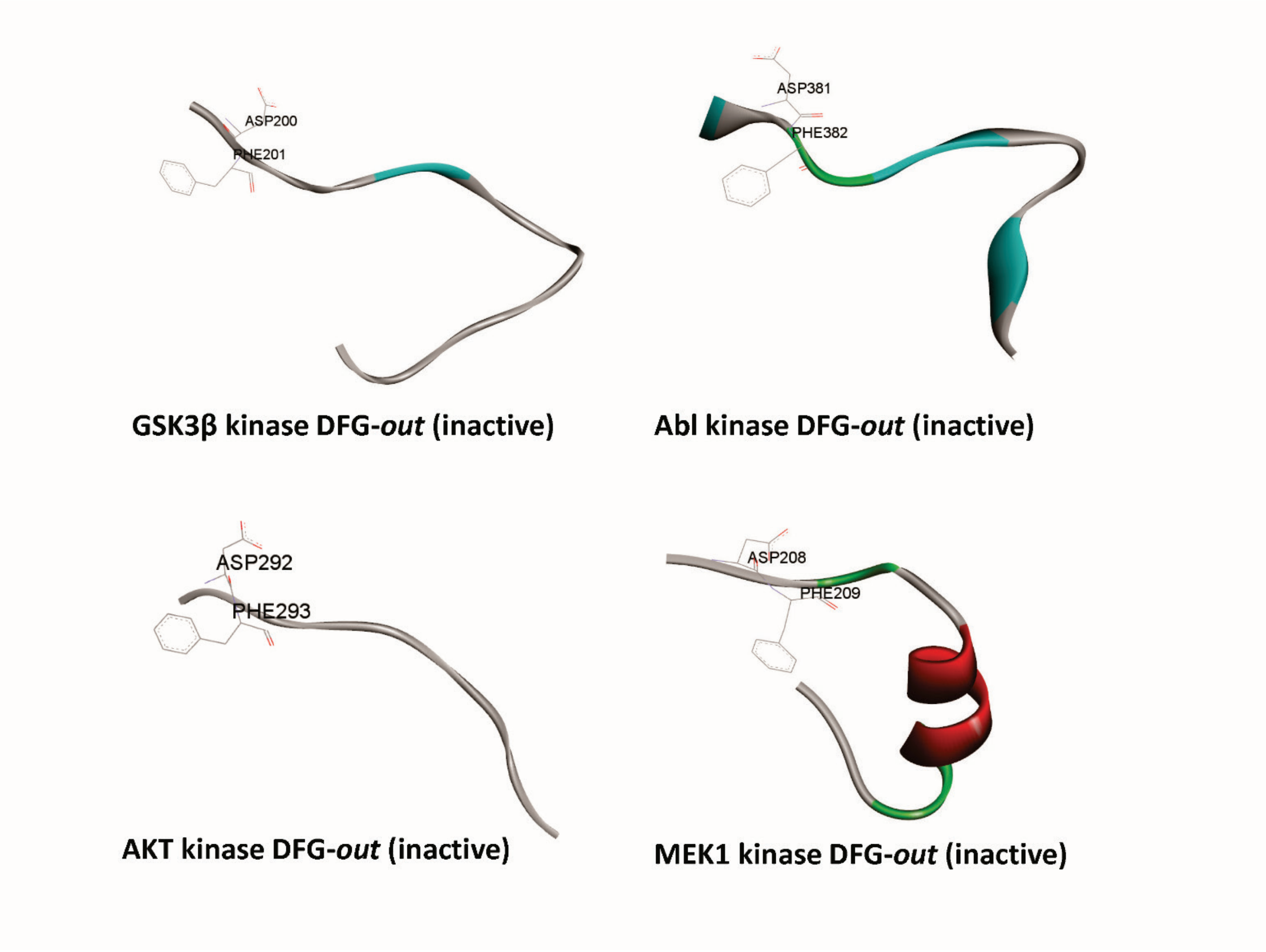

Balasubramaniam et al., Supplementary Figure 2

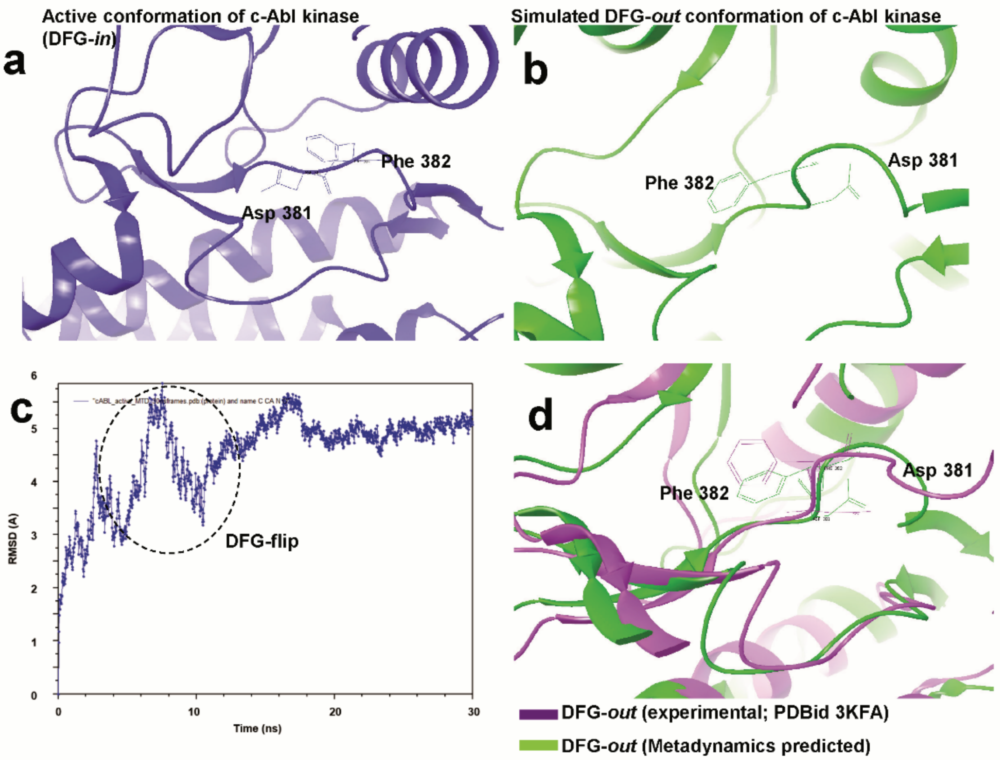

Balasubramaniam et al., Supplementary Figure 3

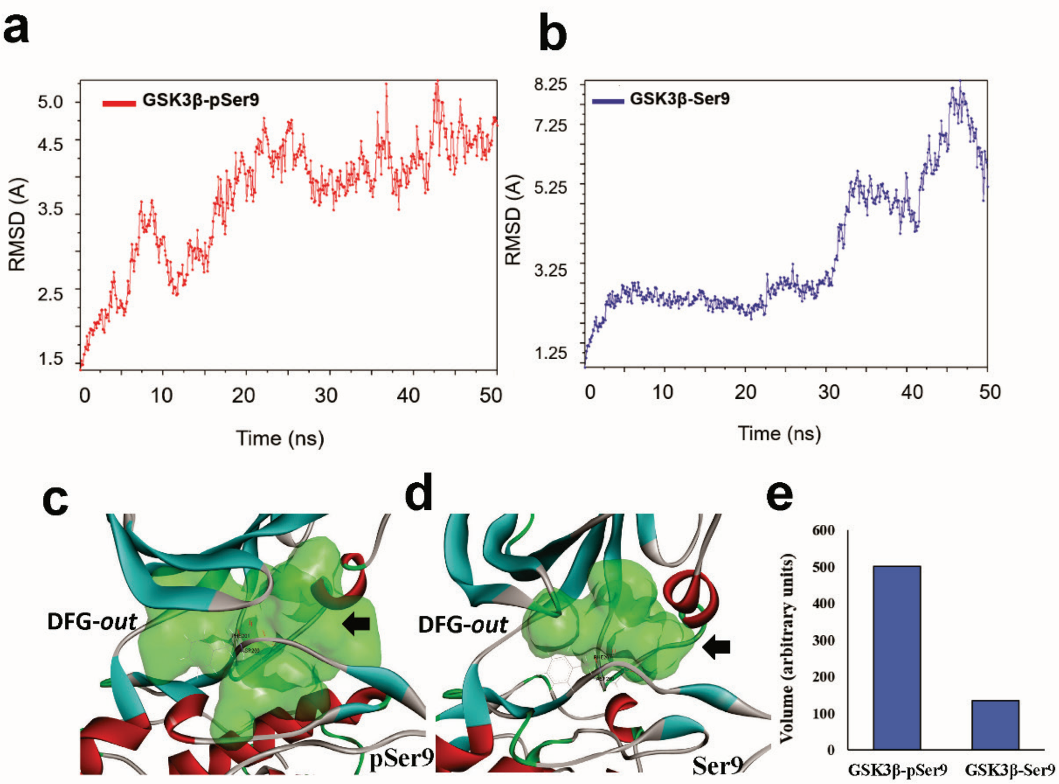

Balasubramaniam et al., Supplementary Figure 4

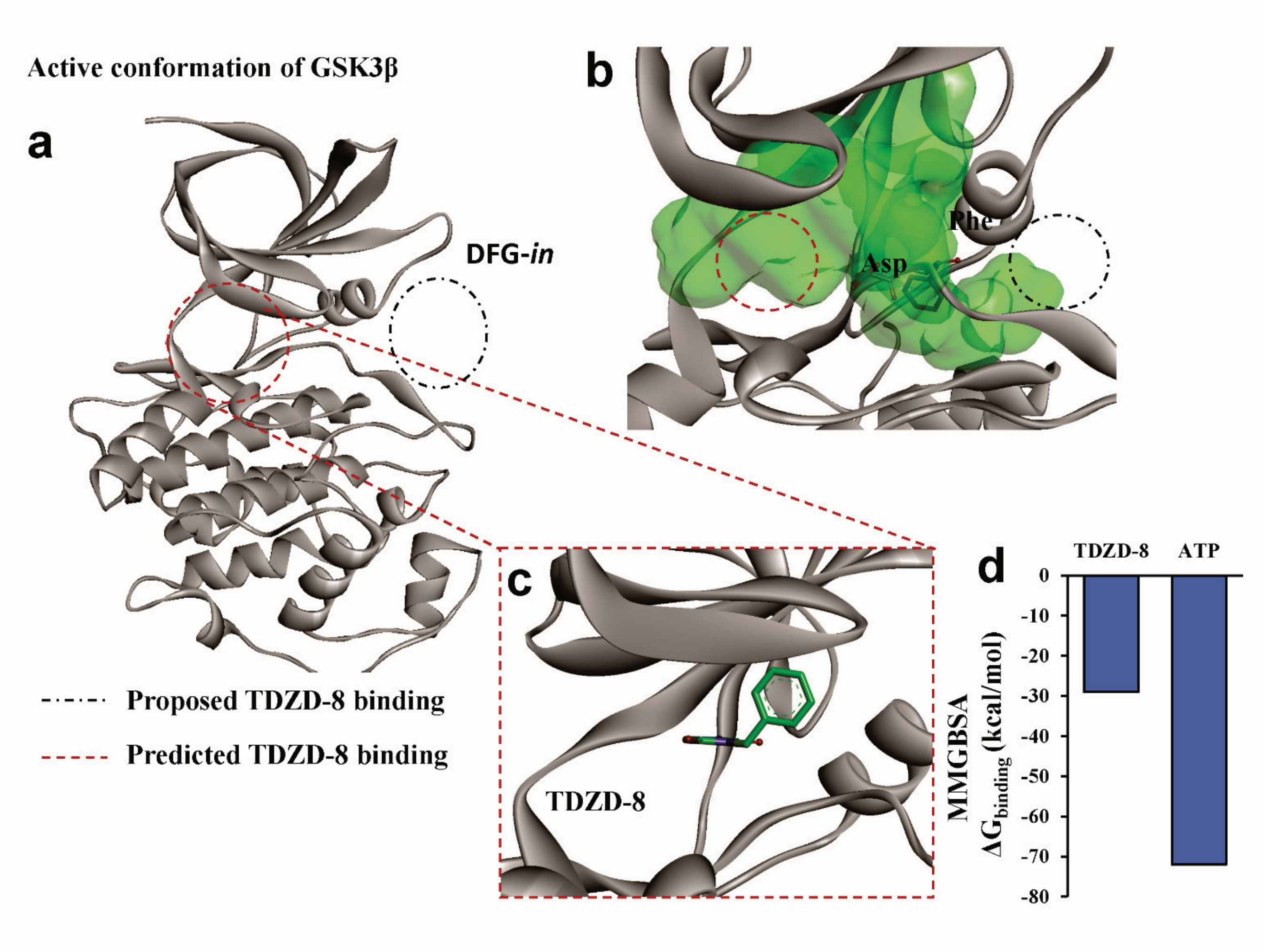

Balasubramaniam et al., Supplementary Figure 5

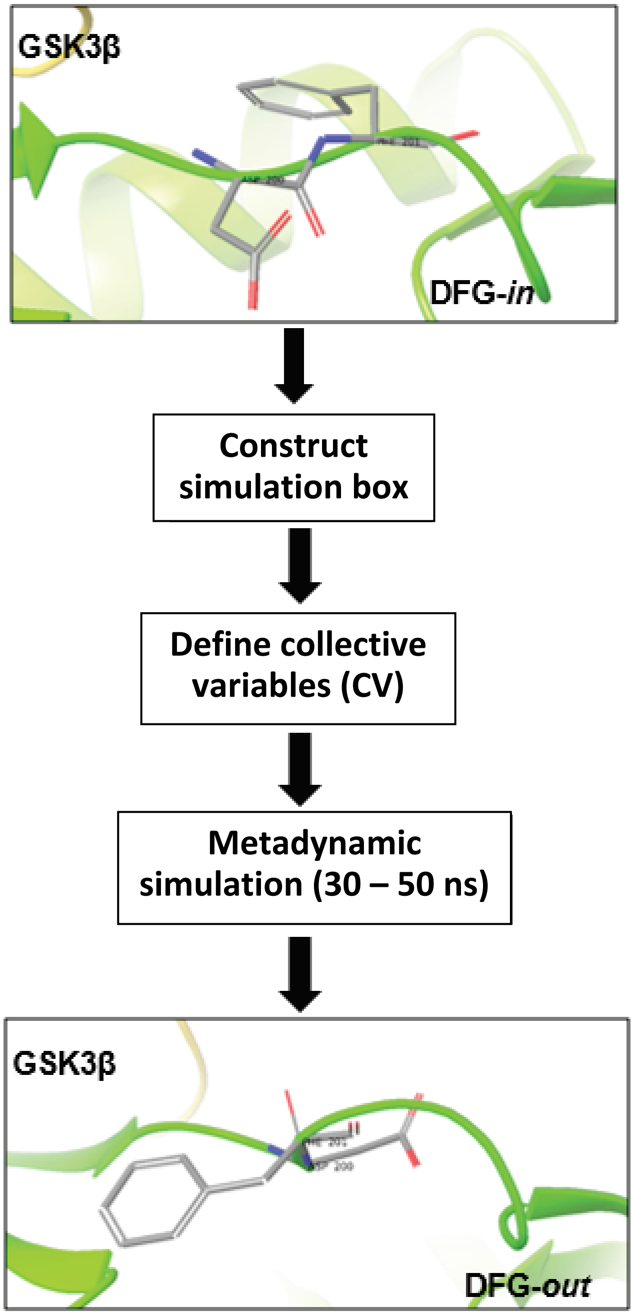

Supplement: Supplementary file 1 — Supplementary Information 1. [file 41598_2020_75020_MOESM1_ESM.pdf]
